# Astrocytic Acid-Sensing Ion Channel 1a Contributes to the Development of Epileptic Cognitive Impairment

**DOI:** 10.3390/biom15010142

**Published:** 2025-01-17

**Authors:** Wen Li, Huimin Zhou, Xiaona Li, Gengyao Hu, Dong Wei

**Affiliations:** Department of Neurology, Xijing Hospital, Fourth Military Medical University, Xi’an 710032, China; wwyy0929@126.com (W.L.); zhouhuimin@fmmu.edu.cn (H.Z.); xndwyyx2022@126.com (X.L.); 304517021@fmmu.edu.cn (G.H.)

**Keywords:** ASIC1a, cognitive impairment, epilepsy, intracellular Ca^2^, glutamate glial neurotransmitter receptors, synaptic proteins

## Abstract

Reactive astrogliosis and acidosis, common features of epileptogenic lesions, express a high level of astrocytic acid-sensing ion channel-1a (ASIC1a), a proton-gated cation channel and key mediator of responses to neuronal injury. This study investigates the role of astrocytic ASIC1a in cognitive impairment following epilepsy. Status epilepticus (SE) in C57/BL6 mice was induced using lithium–pilocarpine; the impact of ASIC1a on astrocytes was assessed using rAAV–ASIC1a–NC and rAAV–ASIC1a–shRNA, injected in the CA3 region of mice. Behavioral assessments were conducted using the Morris water maze (MWM). Western blotting and immunofluorescence were applied to evaluate ASIC1a and *Gfap* expression while analyzing intracellular calcium and extracellular glutamate (Glu) concentrations in primary cultured astrocytes isolated from the brains of 1 to 3-day-old mice and treated LPS. Results showed enhanced astrocyte proliferation and ASIC1a expression in the dentate gyrus of epileptic mice 7, 21, and 28 days post-SE (all *p* < 0.05). Escape latency in the MWM further suggested that ASIC1a regulates cognitive function in mice with chronic epilepsy. LPS stimulation in vitro mimicked inflammatory responses, increasing ASIC1a after 24 h, which increased the concentration of intracellular calcium and extracellular expression of Glu; inhibition of ASIC1a expression reversed this process. To sum up, these data confirm that astrocytic ASIC1a may facilitate cognitive dysfunction post-epilepsy, presenting a potential therapeutic target.

## 1. Background

Epilepsy is a chronic brain disorder characterized by an enduring predisposition to seizures and by the neurobiological, cognitive, psychological, and social consequences of seizure recurrences [1]. Epilepsy accounts for a significant proportion of the world’s disease burden, affecting around 69 million people worldwide [2]. It affects people of all ages, races, social classes, and geographical locations. Cognitive deficits commonly associated with epilepsy, such as memory impairments, attention deficits, and learning difficulties, are also very common in adult epileptic patients and can cause long-term consequences. For example, studies have suggested that people with epilepsy may have a tough time remembering facts, words, and even things that have happened to them [3]. Other studies have found that patients with epilepsy may have permanent learning disorders caused by a brain lesion and/or a stable brain dysfunction [4]. In addition, studies have suggested that various interlinked factors, including the intensity and duration of seizures, the frequency and early onset of epilepsy, and the anti-epileptic drug treatment, influence most cognitive problems [5,6,7]. Yet, the mechanisms underlying seizure-associated nervous dysfunctions are still not fully understood. Therefore, searching for new therapeutic targets for epilepsy and its related comorbidities is of essential importance.

Astrocytes are complex and highly heterogeneous cells capable of regulating synaptic strength, which are important in regulating cognition function [8]. Once the intracytoplasmic calcium concentration of astrocytes increases, glial transmitters are released, which then regulate the plasticity of synaptic transmission [9]. Some studies have found that astrocytic dysfunction contributes to pathological changes in synaptic transmission and hyperexcitability in epilepsy [10]. Moreover, a recent study suggested that astrocyte-mediated inflammation can promote epileptogenesis and seizure recurrence, especially when endogenous anti-inflammatory molecules fail to resolve inflammation [11]. Thus, monitoring astrocyte activation states could form the basis of prognostic and diagnostic markers of epilepsy [11].

Reactive astrocytes are astrocytes that undergo molecular, morphological, and functional changes in response to pathological situations in the surrounding tissue. They express a high level of astrocytic acid-sensing ion channel-1a (ASIC1a), a proton-gated cation channel and key mediator of responses to neuronal injury [12]. ASICs exhibit unique patterns of distribution in the brain. Over the years, ASIC1a has become a particularly interesting candidate for a variety of pathological and physiological processes, including epilepsy [13]. Studies [14] have found that an acidic environment in chronic epilepsy may activate ASIC1a and increase intracellular calcium concentration, releasing glutamate. Excess glutamate release associated with recurrent seizures and observed in chronic epilepsy leads to long-term alterations in normal neuronal signaling and network connectivity [15]. In addition, studies have found that targeting ASICs in individual glial cells is a therapeutic strategy for various conditions [12]. Yang et al. found that ASIC1a contributes to developing chronic epileptogenesis and may be an attractive new target for treating epilepsy [16]. However, there is still uncertainty about the mechanisms underlying astrocytic ASIC1a-induced cognitive impairment after epilepsy.

Studies have found that calcium influx initiated by ASIC1a activation activates other signaling pathways, such as calmodulin-dependent enzymes such as calmodulin kinase (CaMKII) or phospholipase C (PLC) [17], which further implies that ASIC1a activation is a key mediator of cognitive function in this process. In addition, previous studies suggest that ASIC1a may interact with other ion channels, representing a critical component of the underlying pathology. Also, changes in membrane potential due to ASIC1a activation may modulate membrane excitability and signaling by indirectly influencing the activation state of potassium or sodium channels [18].

This study investigated whether astrocytic ASIC1a is involved in cognitive impairment after epilepsy. Our results show that an abnormal expression and activation of ASIC1a in astrocytes after the formation of chronic epilepsy induced by brain injury increases the intracytoplasmic calcium concentration, which leads to the release of glial transmitters from astrocytes and regulates synaptic plasticity to participate in the occurrence of cognitive impairment in epilepsy.

## 2. Methods

### 2.1. Animals and Pilocarpine-Induced SE Model

C57 BL/6 mice (male, aged 8 weeks, 20–25 g, C58BL/6 wild-type) were obtained from the Experimental Animal Center of the Fourth Military Medical University. All the animals were housed in an environment with a temperature of 22 ± 1 °C, relative humidity of 50 ± 5%, and a light/dark cycle of 12/12 h, and given water and food *ad libitum*. All animal studies (including the mice euthanasia procedure) were conducted in compliance with the regulations and guidelines of the Fourth Military Medical University institutional animal care and conducted according to the AAALAC and the IACUC guidelines.

SE model was induced by lithium–pilocarpine as previously described [19,20]. Pilocarpine (130 mg/kg, Sigma, Kawasaki, Japan) was intraperitoneally injected to induce SE. Lithium chloride (350 mg/kg, Sigma) was intraperitoneally administrated 18 h before pilocarpine was given. During modeling, supportive care (food soaked in a 5% glucose solution for 3 days) was given. Mice were closely observed during SE until they returned to a regular chow diet and behavior.

Sixty minutes after SE onset, diazepam (30 mg/kg, jinyao pharmaceutical) was administrated intraperitoneally to stop the seizures. Seizure intensity was observed and evaluated according to the Racine scale; animals showing IV–V seizures [21] were used in the study.

### 2.2. In Vivo Experiments

This study included 2 main experiments, which are as follows: (1) analysis of the expression pattern of astrocytes ASIC1a during epileptogenesis; (2) analysis of the effect of astrocytes ASIC1a, examined by injecting rAAV-ASIC1a-NC, rAAV-ASIC1a-shRNA, and assessing mice behavior using the Morris water maze (*MWM*) test. The in vivo experiment design is shown in Figure S1.

For experiment 1, mice were first divided into 2 groups: the control group (mice that did not undergo SE, *n* = 6) and the SE group (*n* = 24). Of the 24 mice injected with LiCl-pilocarpine, 22 mice developed SE, and 4 mice died during or shortly after SE. The remaining 18 mice were perfused on day 7, 21, and 28 post-SE (*n* = 6 for each time point).

For experiment 2, 60 WT mice were randomly assigned to four groups as follows: the rAAV-ASIC1a-NC group, rAAV-ASIC1a-shRNA group, rAAV-ASIC1a group, and sham-operated group. Before grouping, mice received a virus injection. Briefly, mice were first anesthetized with conventional doses of pentobarbital in a stereotaxic apparatus. The scalp was then carefully cut along the midpoint of both ears, exposing the bregma and skull; the center of the fontanelle triangle was used as the origin coordinate and the direction coordinate of the hippocampus (M: ±2.3 mm, L: −1.8 mm, V: −2.0 mm). After disinfecting the skin, holes were drilled at bilateral positioning points with dental drills, trying carefully not to damage brain tissue. A microsyringe was then inserted, and a 2 μL virus was injected into each site to the desired depth at a speed of 0.5 μL/min. Then, a 10 min injection was performed by infusion pump, after which the needle was gently removed. The wound was cleaned, and the scalp was sutured. Unlike the virus group, the sham group was injected with a physiological saline (2 μL). After surgical recovery, SE modeling was conducted, including in the WT group.

### 2.3. rAAV-Vector Production

A recombined AAV2 vector carrying a 682 bp mouse glial fibrillary acidic protein (*Gfap*, a marker of astrocytes) promoter specifically targeting astrocytes [22] was used for this study. The construction and production of vectors were performed as previously described [16]. The following sequences of shRNA targeting mouse ASIC1a were used: 5′ -TCGACTATGCCTATGAGGTCATTAACTCGAGAATGACCTCATAGGCATAGTCTTTTTT-3′ (bold: shRNA-sense and -antisense strands, respectively; italics: hairpin turn); scrambled shRNA: 5′ -TCGATTCTCCGAACGTGTCACGTTTCAAGAGAACGTGAC11ACGTTCGGAGAATTTTTTG-3′. ASIC1a-shRNA, ASIC1a-scramble, and ASIC1a-cDNA were subcloned into an expression cassette of the mouse *Gfap* promoter. *Egfp* was used as a reporter gene. DNA sequencing identified the recombinant plasmid.

### 2.4. Morris Water Maze

The black circular water pool (diameter 160 cm, height 40 cm, containing water at 25 ± 0.5 °C) was divided into four equally spaced quadrants. A transparent Plexiglas platform (29 cm in height and 10 cm in diameter) was submerged 1 cm below the water surface, hidden in the center of quadrant I. Mice were trained first and randomly placed in one of the four quadrants in the water, facing the wall of the pool. For training, rats were required to find a platform within 60 s; if the time exceeded 60 s, mice were guided onto the platform and allowed to stay there for 20 s. The time required for the rats to find the target platform was recorded each time. Each rat was trained continuously for 4 days, 4 times a day (fixed times each day), with an interval of 20 min. The probe trial started on day 5. The swimming distance and escape latency to the target platform were recorded as indices representing the learning performance. The medium was removed, and the animal was placed randomly in a quadrant outside the original platform quadrant. The time each rat spent in the target quadrant and the entry frequencies into the target quadrant were recorded and analyzed.

### 2.5. Immunofluorescence Staining

Immunofluorescence staining of the brain was performed following a previously reported protocol [23]. Slices of frozen brain tissue were prepared into 30 μM thick continuous coronal sections using a vibratome and incubated with a primary antibody, mouse anti-ASIC1a (Invitrogen, Thermo Fisher, Carlsbad, VT, USA; MA5-27640; 1:100), rabbit anti-GFAP (Dako, Santa Clara, CA, USA; Z0334; 1:2000), mouse anti-Tuij1 (Abcam, Cambridge, UK; AB78078; 1:200), and rabbit anti-PSD95 (Abcam, Cambridge, UK; AB18258; 1:200) at 4 °C overnight. After washing, slices were incubated with a secondary antibody (including 488/594 labeled goat anti-mouse IgG antibody (Invitrogen, Thermo Fisher, Tokyo, Japan, 1:400) and 488/594-labeled goat anti-rabbit IgG (Invitrogen, Thermo Fisher, Tokyo, Japan 1:500)) for 2 h at room temperature. Nuclei were then counterstained with Hoechst 33258 (Solarbio, Beijing, China; C0020;1:2000) for 5 min at room temperature. Slices were finally analyzed using a Nikon microscope.

### 2.6. Immunohistochemistry

Slices of frozen brain tissue were prepared into 30 μm thick continuous coronal sections using a vibratome, incubated in PBS containing 3% H_2_O_2_ (3 × 5 min) to block endogenous peroxidase activity and in PBS containing 1% goat serum albumin to block non-specific binding. Samples were then incubated with mouse anti-ASIC1a primary antibody (Invitrogen, Thermo Fisher, Tokyo, Japan; MA5-27640; 1:100) at 4 °C overnight, washed (3 × 10 min) with PBS, and then incubated with the secondary antibody (HRP-conjugated goat anti-mouse secondary IgG (Abcam, Cambridge, UK; ab6789;1:1000)) for 2 h at room temperature in the dark. HRP was detected using DAB as a substrate, with a Color Developing Reagent Kit (Zhongshan Golden Bridge Biotechnology, Beijing, China). After hematoxylin staining, we observed the images under a Nikon microscope.

### 2.7. Primary Culture of Cortical Astrocytes and in Vitro Experiment

Primary cultures of astrocytes were prepared as previously described [24]. Primary astrocytes were obtained from the brains of 1 to 3 day-old mice (C57BL/6J). Briefly, cells were trypsinized (0.125% trypsin with 0.02% EDTA, 15 min) and neutralized with 1 mL trypsin inhibitor (0.125 mg/mL; Sigma), St. Louis, MO, USA. The cell suspension was then centrifuged (800 rpm, 5 min). Dissociated cells were resuspended in Dulbecco’s Modified Eagle Medium F12 (DMEM/F-12, Invitrogen, Thermo Fisher, Carlsbad, VT, USA) containing 15% fetal bovine serum (FBS) and penicillin 10,000 U/mL/streptomycin 10000 µg/mL (GIBCO) and placed in a humidified atmosphere containing 5%CO_2_/95% air at 37 °C. After 14 days, cultures were shaken at 280 rpm overnight to remove non-astrocytic cells. Purified astrocytes were used for experiments after two passages.

To assess the association between ASIC1a on astrocyte and synaptic regulation, in vitro experiment was conducted. Our previous experiments have shown that ASIC1a is activated after LPS stimulation of primary astrocytes [16]. In this study, the nonspecific inhibitor of ASIC1a was used to validate the experiment. First, primary astrocytes were stimulated with LPS (2 μg/mL, Sigma), after which the cells were incubated with MPEP (mGluR inhibitor), TMB-8 (intracellular calcium release inhibitor), or amiloride; PBS was used for a control group. Meanwhile, rAAV-ASIC1a, rAAV-ASIC1a-shRNA, and rAAV-empty were designed to explore the relationship between ASIC1a, calcium ions, and glutamate glial neurotransmitter receptors.

### 2.8. Western Blot

Cells were lysed in the prepared RIPA cell lysis buffer (Beyotime, Beyotime Biotechnology, Shanghai, China, P0013C). Protein quantification was performed using the Beyotime reagent kit (Beyotime; P0012S). Proteins were then run on Tricine–SDS-PAGE, transferred onto PVDF (polyvinylidene difluoride) membranes (Millipore, Merck Millipore, Burlington, MA, USA), blocked with 5% skim milk/TBST (0.1% Tween-20, TBS) for 1 h, and then the membrane incubated with mouse anti-ASIC1a (Invitrogen; MA5-45592; 1:500) overnight at 4 °C; rabbit-anti β-actin (Invitrogen, Thermo Fisher, Carlsbad, VT, USA; 15G5A11/E2;1:5000) was used as the loading control. After washing with PBS, the bands were incubated with the appropriate secondary antibody (HRP-conjugated) for 1 h at room temperature. An advanced ECL detection kit (Millipore, Merck Millipore, Burlington, MA, USA) was used to detect the band. Images were captured and digitally analyzed using the Bio-Rad System. Band intensities were quantified by Image-ProPlus 6.0 software.

### 2.9. Real-Time PCR

The total RNA was isolated from the primary astrocytes using the Trizol reagent (BioTeke, Beijing, China). Total RNA was first treated with DNase I (ThermoFisher, Waltham, MA, USA). After reverse transcription, PCR products were detected and analyzed using SYBR Green PCR Master Mix (TaKaRa, Beijing, China) during PCR cycles and product aggregation. The specific sequences of primers for the rat ASIC1a and β-actin were as follows: ASIC1a, 5′-AAAGTGGCGGTAGATGGTA-3′ and 5′-AGGAAGACTGATGGCTGAA-3′; β-actin, 5′-CTGTGCCCATCTACGAGGGCTAT-3′ and 5′-TTTGATGTCACGCACGATTTCC-3. Amplification conditions were as follows: one cycle of 94 °C for 5 min, 94 °C 10 s, 60 °C 20 s, 72 °C 30 s for an additional 40 cycles. β-actin was used to normalize the relative mRNA expression of a target gene.

### 2.10. Calcium and Glu Imaging and Colorimetric Method

Calcium imaging was performed as described previously [25]. Briefly, cells were incubated at a 24-well culture plate for 30 min at 37 °C with 5 μM Fura-4 (Sigma-Aldrich, St. Louis, MO, USA) in PBS after cultured astrocytes were pretreated with LPS (2 μg/mL) for 24 h. Fura-4 fluorescence was measured using 494 nm excitation and 510 nm emission. Cells were positioned on an inverted optics microscope (Olympus, Tokyo, Japan, FV1000). Digitized images were acquired and analyzed by FV10-ASW 3.1. Averaging pixel ratio values in circumscribed regions of cells in the field of view were recorded, and ratio images were analyzed using Origin 8.0 for further analysis.

Glu expression was evaluated by the colorimetric method (BC1585, Solarbio, Beijing, China). The test is based on converting glutamate and NAD to α-Ketoglutaric acid, NADH, and NH4+ catalyzed by glutamate dehydrogenase (GDH). This catalytic reaction causes an increase in absorbance at 340 nm. The glutamate content can be calculated by measuring the change in absorbance at 340 nm.

### 2.11. Statistical Analysis

All data, expressed as average ± standard error, were analyzed by a *t*-test provided by GraphPad Prism 7 software. Comparisons between two groups were made using a t-test; comparisons between three groups were made using ANOVA. All *p*-values were two-sided, and *p* < 0.05 was considered statistically significant.

## 3. Results

### 3.1. Epileptic Mice Show Proliferation of Astrocytes and High Levels of ASIC1a Expression

**Figure 1:**
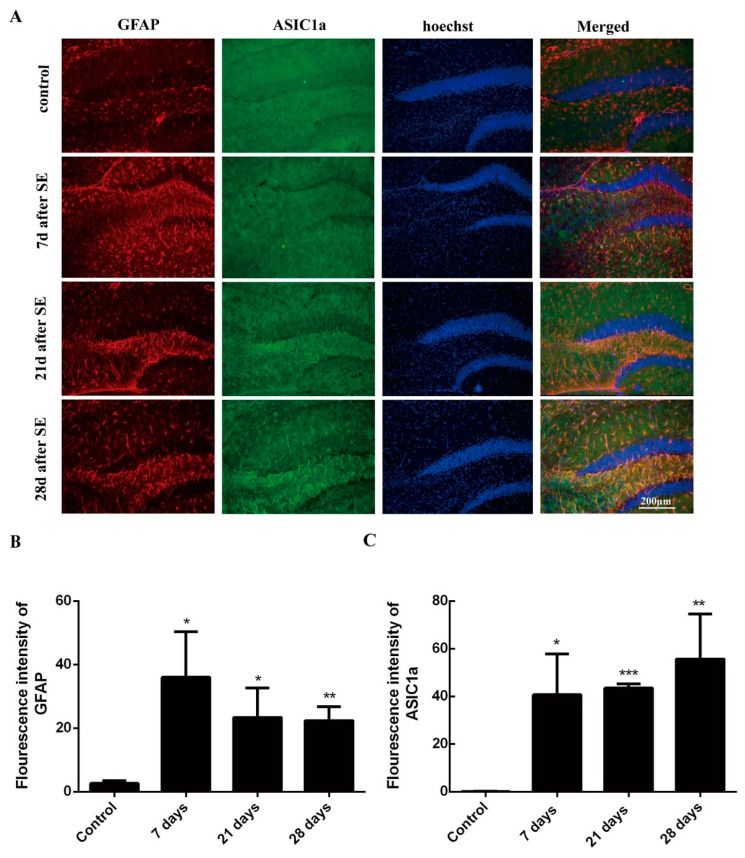
Massive expression of ASIC1a in hippocampal astrocytes of epileptic mice. (**A**) Immunofluorescence staining was performed on day 7, 21, and 28 post-SE. ASIC1a (shown in green), GFAP (shown in red), and Hoechst (shown in blue). (**B**) The fraction of astrocyte expression between day 7, 21, and 28 post-SE. * *p* <0.05, ** *p* < 0.01. (**C**) ASIC1a expression in hippocampal astrocytes increased on day 7, 21, and 28 days post-SE (unpaired *t*-test). * *p* <0.05, ** *p* < 0.01, *** *p* < 0.001.

Previous studies reported that astrocytes proliferate after epilepsy [26,27]; yet the exact mechanism of action that affects cognitive ability is still not fully explored. In this study, we further assessed the expression changes of the glial fibrillary acidic protein (*Gfap*) and ASIC1a in mice brains after SE (Figure 1A). *Gfap* is considered one of the best markers for assessing activating astrocytes following injury or stress in the central nervous system [28]. Seven days after modeling, the number of astrocytes (i.e., *Gfap* staining positive) in epileptic mice increased in the dentate gyrus compared to the control mice (*p* < 0.001, Figure 1B,C) but then decreased slightly on day 21 and 28 post-modeling (all *p* < 0.05), which suggests astrocytes proliferate after SE.

Our previous study [16] confirmed that ASIC1a is activated during astrocytes proliferation. In this study, ASIC1a expression increased from day 7 to day 28 compared to the control mice (*p* < 0.01, Figure 1B,C), which further suggests that ASIC1a is activated in astrocytes after SE.

### 3.2. Overexpression of ASIC1a in Primary Cultured Astrocytes Increases the Concentration of Intracellular Calcium and Extracellular Expression of Glu

**Figure 2:**
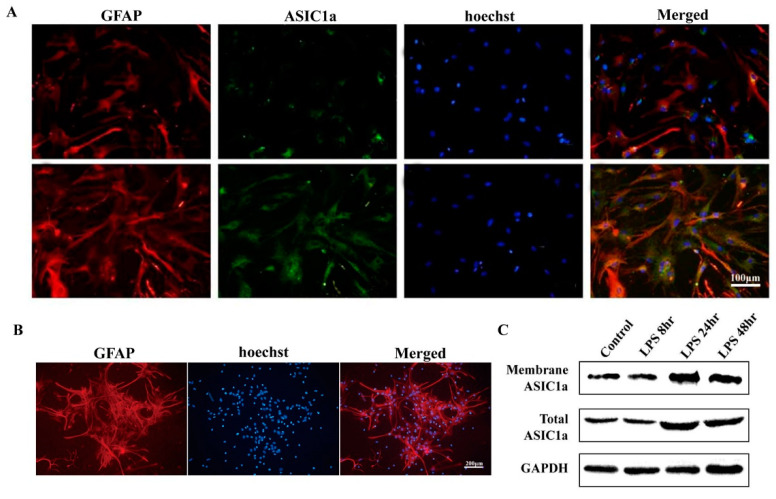
LPS stimulation can increase the expression of ASIC1a on primary astrocytes. (**A**) Immunofluorescence staining was performed on cultured astrocytes treated with LPS, which were compared to control cells. ASIC1a (shown in red) and GFAP (shown in green). (**B**) *Gfap* (shown in red) immunofluorescence staining of astrocytes. (**C**) Membrane and total ASIC1a protein in control and LPS-treated astrocytes on 8, 24, and 48 h after treatment assessed using Western blot.

LPS may activate inflammatory-reactive astrocytes in vitro [29]. In this study, primary cultured astrocytes were isolated from the brains of 1 to 3 day-old mice (C57BL/6) following a previously reported approach [30] and then treated with LPS (2 μg/mL) for 24 h. All cells showed positive staining for *Gfap* (Figure 2A). We found that ASIC1a was highly expressed in astrocytes after LPS stimulation, which was further confirmed by immunofluorescence and Western Blot (Figure 2B,C).

In this study, we found that ASIC1a was activated under tissue acidification conditions, causing extracellular calcium influx, which further confirms previous studies [17], which suggested that ASIC1a is sensitive to transient acidification responses. ASIC1a is an acid-sensitive ion channel, and an acidic environment can stimulate the opening of ion channels [31]. Also, lactate accumulation after inflammation causes tissue acidification [32]. Rapid acidification due to seizures may activate ASIC1a in the early stages, triggering a cascade of intracellular calcium influx and neurotransmitter release responses, resulting in abnormal synaptic transmission. In the long term, this persistent acidifying environment may lead to ASIC1a inactivation, which is reflected in the continued decline in cognitive function [14].

**Figure 3:**
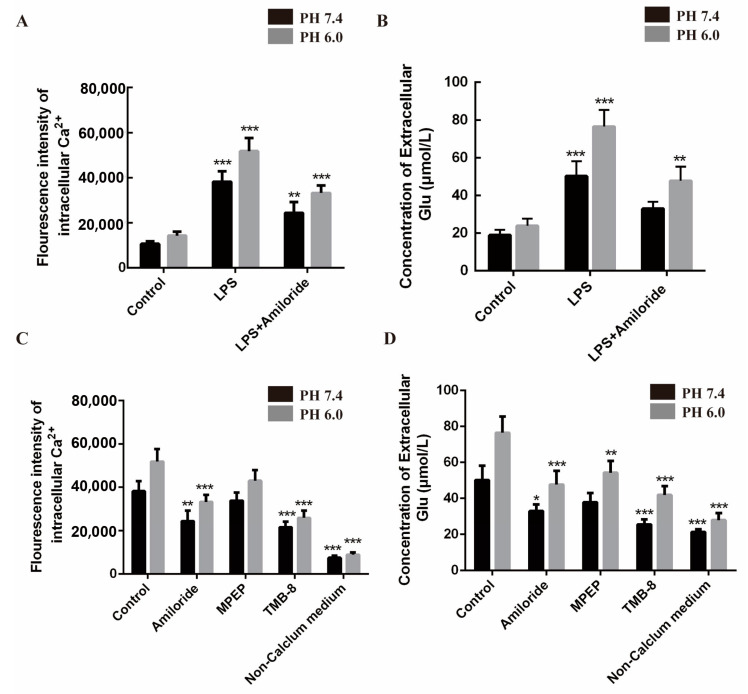
Increased expression of astrocytic ASIC1a mediated calcium elevation and glutamate receptor subunit expression after LPS-treated astrocytes. (**A**,**B**) Calcium ion imaging technology and glutamate concentration detection performed on cultured astrocytes. Representative changes of intracellular calcium concentration and extracellular Glu in response to extracellular pH reduction (from 7.4 to 6.0) in the absence and presence of amiloride. The fluorescence intensity of intracellular calcium and the concentration of extracellular Glu markedly increased 24 h after LPS treatment, compared with the control group. This phenomenon can be inhibited by amiloride. ** *p* < 0.01, *** *p* < 0.001. (**C**,**D**) Amiloride effected the calcium intensity and Glu concentration in LPS-treated astrocytes: Asic1a: nonspecific inhibitor; MPEP glutamate receptor inhibitor; TMB-8: inhibitors of intracellular calcium release; non-calcium extracellular fluid. * *p* < 0.05, ** *p* < 0.01, *** *p* < 0.001.

To further verify whether the increase in intracellular calcium concentration will lead to the release of glial transmitters, primary cultured astrocytes were grouped as follows: control group, LPS group, and LPS + amiloride group. Amiloride is an ASIC1a-nonspecific antagonist [33] and blocker of Na^+^/H^+^ exchange [34]. LPS stimulation increased intracellular calcium concentration and extracellular Glu for 24 h (pH was reduced from 7.4 to 7.0) compared to control cells (Figure 3A,B). However, this phenomenon was inhibited by amiloride (Figure 3A,B). These data suggest that the expression of ASIC1a in astrocytes increases the concentration of intracellular calcium and the extracellular expression of Glu, while ASIC1a expression inhibition may reverse this action.

In order to further investigate the interaction of ASIC1a, intracellular calcium concentration, and extracellular expression of Glu, we treated astrocytes with MPEP (mGluR inhibitor), TMB-8 (intracellular calcium release inhibitor), and amiloride. The role of ASIC1a was determined by using MEPE, TMB-8, amiloride, and extracellular fluid without Ca^2+^. We detected intracellular calcium concentration and extracellular Glu transmitter concentration under different pH conditions, and LPS-treated astrocytes were set as a control group. Intracellular calcium concentration and extracellular Glu concentration decreased in astrocytes cultured with amiloride. Only Glu concentration was reduced when the mGluR inhibitor MEPE was added, but there was no significant difference in calcium concentration. However, calcium inhibitor TMB-8 promoted the significant reduction in both intracellular calcium concentration and extracellular Glu concentration; this phenomenon occurred again when there was no calcium in the culture medium, compared with the control group (Figure 3C,D).

### 3.3. A Direct Promotion Effect of ASIC1a on Intracellular Calcium Expression and Extracellular Glu Using the Salvage Experiment

**Figure 4:**
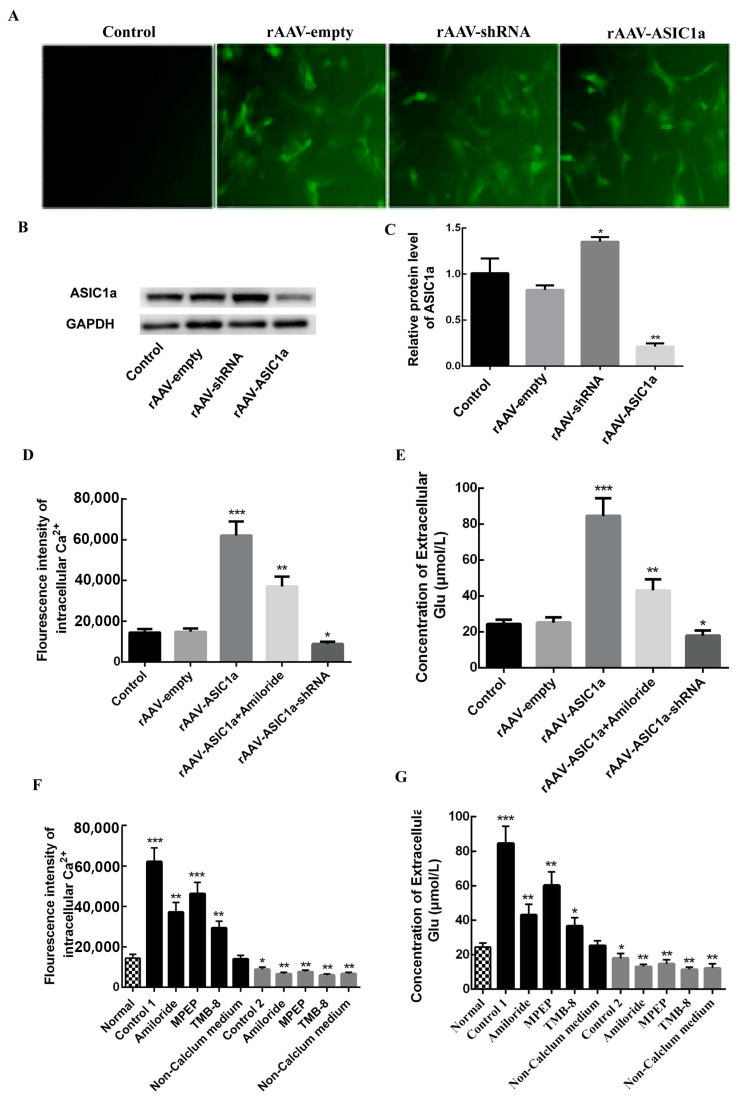
rAAV adenovirus induced calcium concentration and extracellular Glu expression in cultured astrocytes. (**A**,**B**) After virus transduction, calcium concentration and extracellular Glu expression increased or decreased, accompanied by the expression of ASIC1a. rAAV-ASIC1a significantly improved calcium concentration and extracellular Glu expression, while rAAV-ASIC1a-shRNA virus significantly reduced calcium concentration and extracellular Glu expression. * *p* < 0.01, ** *p* < 0.001. (**C**–**G**) rAAV-ASIC1a virus transduction in cultured astrocytes (control 1); rAAV-ASIC1a-shRNA virus transduction in cultured astrocytes (control 2). Amiloride, MPEP, TMB-8, and non-calcium medium markedly decreased calcium concentration and extracellular Glu expression. * *p* < 0.05, ** *p* < 0.01, *** *p* < 0.001. After knocking down the expression of ASIC1a, only TMB-8 reduced calcium concentration and extracellular Glu expression.

Next, we assessed a direct promotion effect of ASIC1a on intracellular calcium expression and extracellular Glu using the salvage experiment. First, we constructed the overexpression and knockdown AAV virus system of ASIC1a using rAAV-ASIC1a and rAAV-ASIC1a-shRNA. The design of the sequence refers to our previous research [16]. A successful inhibition or upregulation of ASIC1a was further validated, as shown in (Figure 4A,C).

Consequently, we assessed the concentration of intracellular calcium and glutamate receptor subunit expression in astrocytes overexpressing or downregulating ASIC1a, which were then treated with LPS, LPS + amiloride, LPS, amiloride, MPEP, TMB, and non-calcium extracellular fluid (Figure 4D). Both extracellular calcium and Glu increased in astrocytes overexpressing ASIC1a, while they decreased when ASIC1a was inhibited (Figure 4D,E). Moreover, amiloride reversed this process. MPEP, TMB-8, and non-calcium medium markedly decreased calcium concentration and extracellular Glu expression in cells overexpressing or downregulating ASIC1a (Figure 4F,G).

### 3.4. The Role of ASIC1a in Cognitive Function in Mice with Chronic Epilepsy

**Figure 5:**
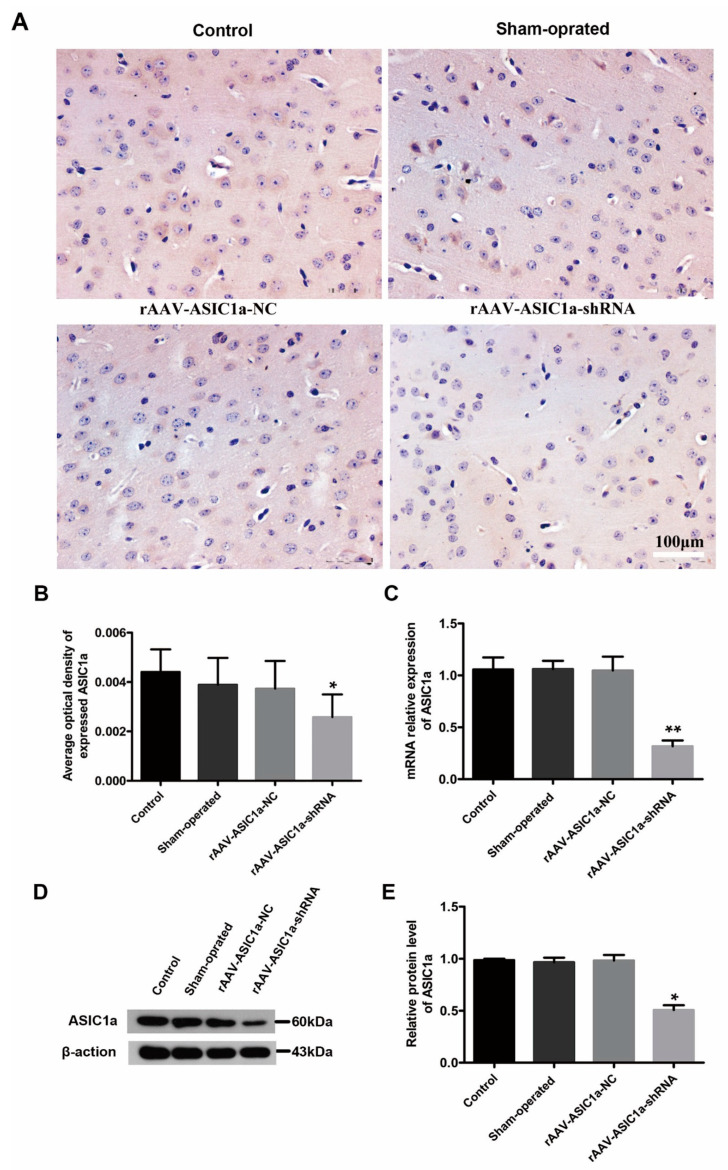
ASIC1a expression was efficiently induced by viral vector at 4 weeks after SE. (**A**) Immunohistochemical staining of ASIC1a in each group. (**B**–**E**) Average optical density of expression ASIC1a. ASIC1a mRNA expression and protein level were measured using fluorescence quantitative analysis, RT-PCR, and Western blot. * *p* < 0.05, ** *p* < 0.01.

In order to verify the function of ASIC1a, we further carried out the function verification experiment of ASIC1a in vivo. WT mice were randomly divided into 4 groups: rAAV-ASIC1a-NC group, rAAV-ASIC1a-shRNA group, rAAV-ASIC1a group, and sham-operated group. rAAV- GFAP-ASIC1a-shRNA could effectively knock down the expression of ASIC1a in mouse brain tissue, which was confirmed by immunohistochemistry, Western blot, and RT-PCR experiments (all *p* < 0.05) (Figure 5).

**Figure 6:**
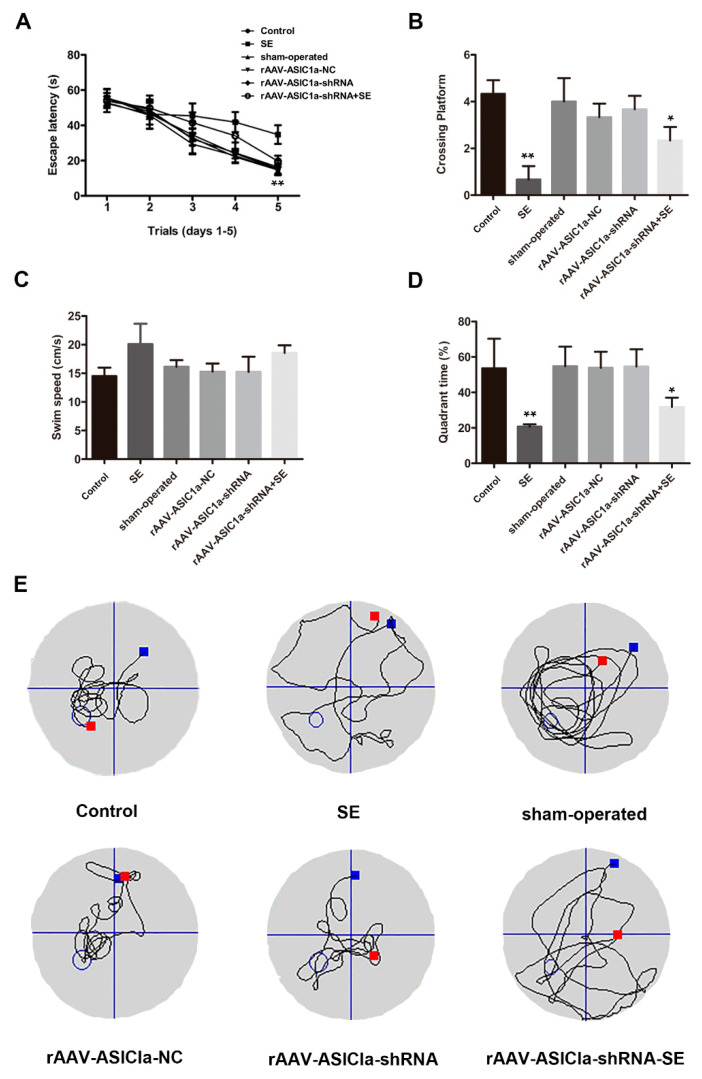
Knocking down the expression of ASIC1A can improve cognitive function in mice. (**A**) The escape latency in the passive avoidance test was calculated in each group (Mean ± SEM). ** *p* < 0.01. (**B**) The percentage within the target quadrant in space detection tests. Mean ± SEM; * *p* < 0.05 and **p < 0.01. (**C**) Calculate the swimming speed for each set. (**D**) The target quadrant percentage is the number of platform quadrant crossings in the spatial probe test. (Mean ± SEM), * *p* < 0.05; ** *p* < 0.01. (**E**) Representative traces of each group. Red is transparent Plexiglas platform. Blue is trarget platform.

Then, we conducted water maze experiments on mice to detect cognitive function. The escape latency markedly declined over the training days in all groups. The escape latency in the WT mice was longer than that in rAAV-GFAP-ASIC1a-shRNA mice after SE (Figure 6A); significant differences were observed between the control group, sham-operated group, rAAV-ASIC1a-NC group, and rAAV-ASIC1a-shRNA group (all *p* < 0.05). After SE molding, the ASIC1a expression increased while the learning ability was reduced compared to the WT group (Figure 6B). During the spatial probe test, the number of platform crossings was significantly higher in the WT mice group compared with the rAAV-ASIC1a-shRNA-infected group after SE. Furthermore, the rAAV-ASIC1a-shRNA infected after the SE group traveled a shorter distance (13.44 ± 3.79) and spent more time (31.79 ± 5.22) in the target quadrant.

In contrast, WT mice after the SE group traveled a longer distance (40.87 ± 5.29) and spent more time (20.72 ± 1.34), while there was no difference in other detected groups (Figure 6C–E). These data suggest that ASIC1a regulates cognitive function in mice with chronic epilepsy.

## 4. Discussion

Epilepsy is the most common major neurological disorder associated with cognitive and behavioral impairments [5]. However, the mechanism leading to cognitive impairment after epilepsy is still not fully understood. Therefore, it is important to characterize the underlying mechanisms of cognitive impairment after epilepsy, as this could lead to novel interventions that would ultimately improve the quality of life. The present study assessed the role of ASIC1a, a proton-gated cation channel and key mediator of responses to neuronal injury, on cognitive impairment after epilepsy. We used a lithium–pilocarpine to induce an animal model of epilepsy, a popular model of acute SE and chronic temporal lobe epilepsy [35], finding that astrocyte proliferation and increased expression of ASIC1a may be one of the pathological mechanisms of cognitive dysfunction after epilepsy.

Astrocytes are the most abundant type of glial cells in the mammalian brain that play an important role in encephalopathy by regulating the blood–brain barrier (BBB) integrity, modulation of synaptic transmission, and regulation of ion homeostasis [36]. Recent studies have shown that astrocytes are important in regulating cognitive function [37]. The accepted molecular basis of cognitive function is that the increased calcium concentration in astrocytes leads to their release of glial transmitters, thus regulating the plasticity of synaptic transmission [38]. Astrocytic calcium signaling is pivotal in the communication between astrocytes and neurons [39,40]. Also, evidence indicates that activation of calcium signals in astrocytes induces the subsequent release of gliotransmitters, which may amplify the propensity for epileptic seizures. The present study found that ASIC1a activation in reactive astrocytes significantly elevated cytosolic calcium levels. Also, glutamate levels increased at the same time in vivo and in vitro. These data suggest that changes in intracellular calcium concentration and extracellular Glu concentration accompany the changes in ASIC1a expression. Thus, these data imply that the control of ASIC1a activity may be an alternative strategy to reduce the damage to neurons and cognitive function by acidification, especially in the early stages of epilepsy.

ASICs are activated by the extracellular acidification calcium channel. ENaC/DEG family members have four coding genes: *ASIC1*, *ASIC2*, *ASIC3*, and *ASIC4*. These four genes encode seven subunit proteins, i.e., ASIC1a, ASIC1b, ASIC1b2, ASIC2a, ASIC2b, ASIC3, and ASIC4, which are highly permeable to sodium [41]. ASIC1a is a member of a novel family of proton-gated-amiloride-sensitive cation channels expressed primarily in calcium-permeable neurons [42]. Activation of these channels results in intracellular calcium accumulation, which is important in neurological disorders such as brain ischemia, multiple sclerosis, and spinal cord injury [43,44]. In the physiological state, ASIC1a is mainly expressed in neurons [45]. This study found that ASIC1a is abnormally expressed in many astrocytes activated during epilepsy. This topic takes this clue as a starting point to study the role of ASIC1a on astrocytes in cognitive function impairment after epilepsy.

Although the current study has yielded some important findings regarding ASIC1a and its role in cognitive impairment after epilepsy, several limitations should be noted. First, it is an exciting observation that ASIC1a directly influences synaptic plasticity. Further studies on the effect of ASIC1a on the electrophysiological characteristics of neurons are expected to provide more information. Second, although LPS stimulation can cause an increase in ASIC1a expression in astrocytes in vitro, it cannot fully reproduce the complex inflammatory microenvironment in vivo. For example, inflammatory processes in vivo involve a variety of immune cells, cytokines, and a persistent acidic environment that may be simplified or missing in in vitro models. Thus, further in vivo studies are needed to elucidate the deeper underlying mechanism of cognitive impairment after epilepsy induced by astrocytic ASIC1a activation. Also, more research is needed to further elucidate the mechanisms through which ASIC1a influences behavior and neuron physiology.

## 5. Conclusions

**Figure 7:**
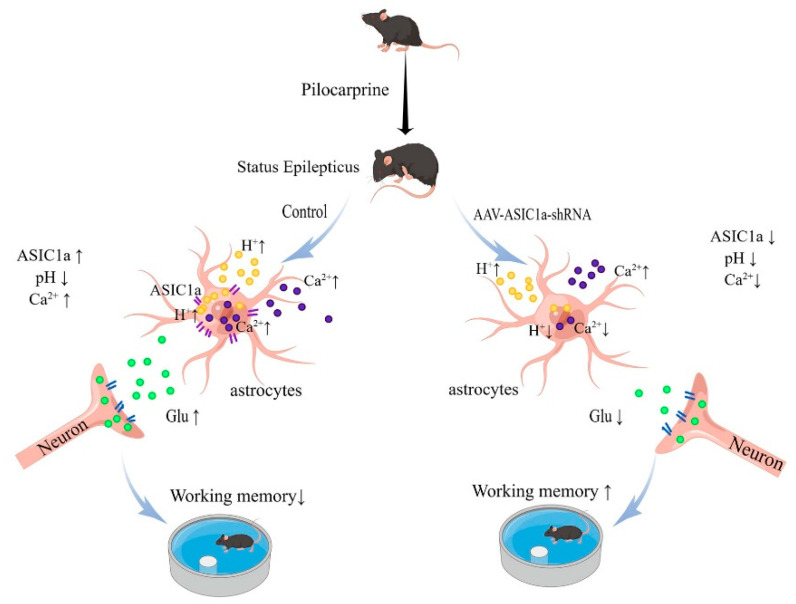
Proposed role of astrocytic ASIC1a in the development of chronic after epilepsy. Epilepticus can cause hippocampal sclerosis. Reactive astrogliosis and tissue acidosis are the prominent features in sclerotic hippocampi. Reactive astrocytes express a high level of ASIC1a, which can be activated by local extracellular low pH. This leads to excessive calcium influx in astrocytes and release of gliotransmitters, and, in turn, affects cognitive function.

Our data suggest that astrocyte proliferation and increased expression of ASIC1a may be one of the pathological mechanisms of cognitive dysfunction after epilepsy. ASIC1a can mediate intracellular calcium elevation and extracellular Glu levels, thus providing a new therapeutic target in cognitive impairment after epilepsy treatment (Figure 7).

## Data Availability

All study results are available and that raw data are available from the corresponding authors.

## Supplementary Materials

The following supporting information can be downloaded at: https://www.mdpi.com/article/10.3390/biom15010142/s1, Figure S1: Flow chart of Pilocarpine injection, virus injection and Morris water maze monitoring, and sampling time point of histological staining.

## List of Abbreviations

ASIC1a	Acid-sensing ion channel-1a
Glu	Glutamate
GDH	Glutamate dehydrogenase
ASICs	Acid-sensitive ion channels
