# Supplementary figures and images for: Astrocytic Acid-Sensing Ion Channel 1a Contributes to the Development of Epileptic Cognitive Impairment
Source: Biomolecules. 2025 Jan 17;15(1):142. doi: 10.3390/biom15010142 (PMC11764220; doi:10.3390/biom15010142)

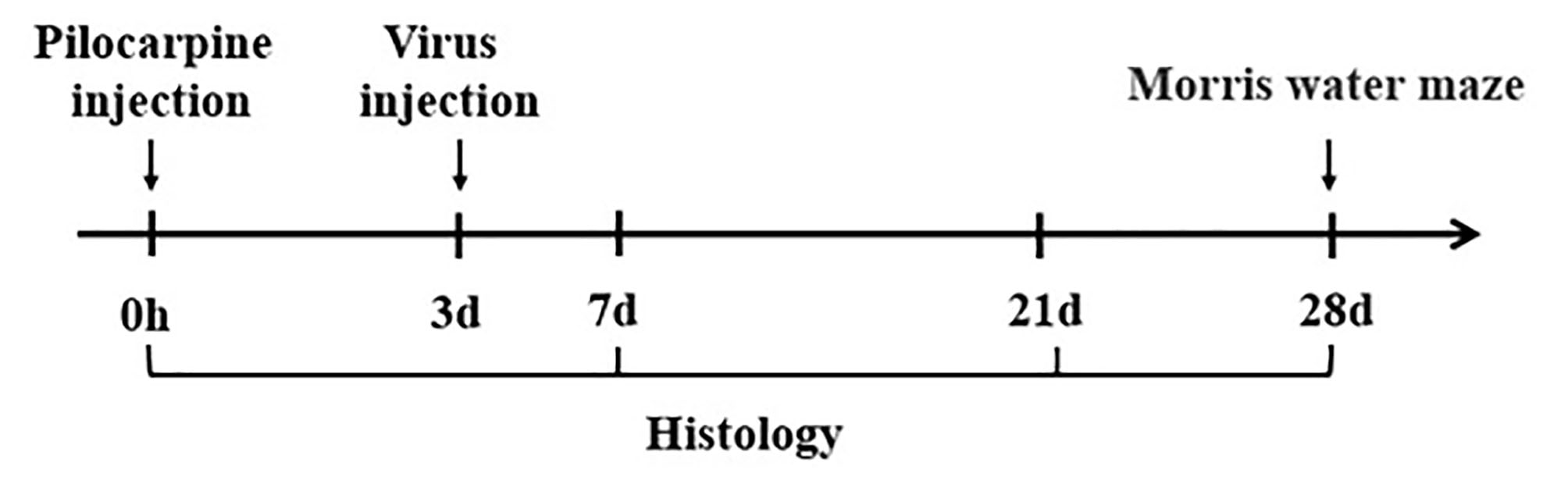

Supplement: Supplementary file 1 [file biomolecules-15-00142-s001.zip › Figure S1.jpg]
