# Supplementary material for: Astrocytic Acid-Sensing Ion Channel 1a Contributes to the Development of Epileptic Cognitive Impairment
Source: Biomolecules. 2025 Jan 17;15(1):142. doi: 10.3390/biom15010142 (PMC11764220; doi:10.3390/biom15010142)
Supplement: Supplementary file 1 [file biomolecules-15-00142-s001.zip › Response to Editor.doc]

Dear Editor:

We really appreciate the critical reading of our manuscript (Manuscript ID: biomolecules-3299031), and we thank you for all the valuable suggestions received from the two reviewers. We have carefully considered the comments and have revised the manuscript accordingly. The responses to the comments are listed one by one as follows (*please see below*).

We are looking forward to hearing about your final decision.

Kind regards

Dong Wei

Department of Neurology, Xijing Hospital, Fourth Military Medical University, China

Email: weieast@fmmu.edu.cn

Point-to-point response

1."Western blotting": we kindly ask you to provide the original image. For all Western blot figures, densitometry readings/intensity ratio of each band should be included; The whole blot showing all the bands with all molecular weight markers on the Western blot should be included. Please send it to me directly via the email attachment.
**Response:** We have compiled the data graphs of all the WB experimental results, and since we have not lost the original data graphs of Figure 2C, we have added a set of experimental data and sent them with the email attachment.

2. Institutional Email: Please provide the institutional emails of all authors except yours. If they do not have institutional emails, please provide their resumes, including research experience and the papers published.
**Response:**

Wen Li: liwen0723.fmmu.edu.cn

Huiming Zhou: zhouhuimin@fmmu.edu.cn

Xiaona Li: xndwyyx2022@126.com

Gengyao Liu: 304517021@fmmu.edu.cn

Dong Wei: weieast@fmmu.edu.cn

3. Authorship: We noticed you have listed three authors as co-first authors, which is not allowed. Please check this and remove one author. Hope for your understanding.
**Response:** Thanks for the reminder, we chose Wen Li# and Huimin Zhou#, two authors as a joint author.

(I) Ensure all references are relevant to the content of the manuscript.

**Response:** Thank you for comment. All ref has been checked. In addition, some new ref has been added.

(II) Highlight any revisions to the manuscript, so editors and reviewers can see any changes made.

**Response:** Thank you for your comment. We have carefully considered the comments and have revised the manuscript accordingly (additional text is shown in red—see revised file). The responses to the comments are listed one by one as follows (*please see below*).

(III) Provide a cover letter to respond to the reviewers’ comments and explain, point by point, the details of the manuscript revisions.

**Response:** Thank you for the suggestion. We have carefully considered the comments and have revised the manuscript accordingly (additional text is shown in red—see revised file). The responses to the comments are listed one by one as follows (*please see below*).

(IV) If the reviewer(s) recommended references, critically analyze them to ensure that their inclusion would enhance your manuscript. If you believe these references are unnecessary, you should not include them.

**Response:** Thank you for your suggestion. We have carefully considered the comments and have revised the manuscript accordingly (additional text is shown in red—see revised file). The responses to the comments are listed one by one as follows (*please see below*).

(V) If you found it impossible to address certain comments in the review reports, include an explanation in your appeal.

**Response:** Thank you for your suggestion. We have carefully considered the comments and have revised the manuscript accordingly (additional text is shown in red—see revised file). The responses to the comments are listed one by one as follows (*please see below*).
